# Comparison of the Algan Hemostatic Agent with Floseal in Rat Liver Laceration Bleeding Model

**DOI:** 10.5152/eurasianjmed.2022.21004

**Published:** 2022-02-01

**Authors:** Kenan Binnetoğlu, Ali Kumandas, Husamettin Ekici, Abdullah Canberk Özbaykuş

**Affiliations:** 1 Department of General Surgery, Kafkas University Faculty of Medicine, Kars, Turkey; 2 Department of Surgery, Kırıkkale University Faculty of Veterinary, Kırıkkale, Turkey; 3 Department of Pharmacology and Toxicology, Kırıkkale University Faculty of Veterinary Medicine, Kırıkkale, Turkey; 4 Department of Medicine Faculty, Bahçeşehir University Faculty of Medicine, İstanbul, Turkey

**Keywords:** Liver laceration, bleeding, Algan hemostatic agent, Floseal, rat

## Abstract

Objective: Major vascular injury is one of the most important causes of death after trauma. The effective and speedy control of the hemorrhage is crucial in reducing deaths. Many products are used for this purpose. Today, however, an ideal product has not yet been produced and there is a strong demand for such effective hemostatic products. The aim of this study is to compare the efficacy of Algan hemostatic agent with Floseal in the liver laceration model in rats.

Materials and Methods: A total of 28 rats were used in the study. Rats were divided into 4 groups, each consisting of 7 rats. Experimental liver laceration was established. In the control group, saline-impregnated gauze was applied. Algan hemostatic agent-impregnated sponge, Algan hemostatic agent powder, and Floseal gel were applied to the experimental groups.

Results: There was no difference in bleeding control among the Algan hemostatic agent powder, Algan hemostatic agent-impregnated sponge, and Floseal. When compared to the control group, Algan hemostatic agent powder, Algan hemostatic agent-impregnated sponge, and Floseal were found to be very effective in bleeding control, respectively (*P* = .001, .012, and .002), in the experimental groups.

Conclusion: This study showed that the properties of both Algan hemostatic agent powder and Algan hemostatic agent-impregnated sponge for controlling bleeding are similar to Floseal. Considering other characteristics such as Algan hemostatic agent’s naturalness, easy applicability, and low cost, Algan hemostatic agent has been a promising effective hemostatic agent.

## Main Points

- The aim of this study is to compare the efficacy of Algan hemostatic agent (AHA) with Floseal in liver laceration model in rats.
- This study showed that AHA had a similar effect to Floseal in controlling bleeding.
- Algan hemostatic agent has given hope as an effective hemostatic agent.

## Introduction

Bleeding is an important cause of death due to trauma.^1-3^ The liver is one of the most commonly injured organs in a trauma. Mortality rate in severe traumatic liver injuries is around 10-15%.^1^ Direct pressure is applied to the bleeding site to stop circulation in trauma patients.^1^ But direct pressure to the bleeding area is not always easy and practical. The fact that some of the deaths caused by bleeding in trauma and surgical operations can be prevented by hemostatic agents has brought intense work in the production of hemostatic agents. Therefore, effective and rapid control of the bleeding is important in reducing deaths. Hence, the early identification and control of bleeding is a crucial step in first aid and post-surgery because intraoperative blood loss increases the incidence of postoperative morbidity and mortality.^4^

Today, hemostatic agents are being produced in different forms and with different mechanisms of action. Some examples are fibrin-based, cellulose-based, gelatin-based, and collagen-based hemostatic agents, sealants (tissue adhesives, such as cyanoacrylates, polyethylene glycol hydrogels, glutaraldehyde-albumina sealants), and combined products. They are already available on the market and are in use in prehospital settings, emergency departments, and operating rooms.^5-7^ Despite the great advances in medicine and the large number of hemostatic agents produced, there is no method other than compression to control external artery bleeding in trauma. Hemostatic agents are mostly in the form of sponges or bandages, and it is important to wrap the bandage tightly or to apply local pressure on the sponge to achieve hemostatic effect. A hemostatic product that will stop arterial bleeding without compression in the bleeding area has yet to be produced. However, in spite of all the major improvements and many products produced for this purpose, an ideal product for bleeding control has not yet been produced despite the strong demand for and effective hemostatic products.

Algan hemostatic agent (AHA) is a herbal extract obtained from the standardized blend of 6 different plants, the first and only patented product in the world. Each of the plants that constitute the AHA subsumes an ingredient that is active either alone or in combination with hemostasis.^8^ The aim of this study is to compare the efficacy of AHA with Floseal as a positive control in the liver laceration model in rats.

## Materials and Methods

### Animals

In the study, 28 rats, all within a weight range of 180-210 g and an age range of 5-7 weeks were used. Rats were fed ad libitum and examined under standard laboratory conditions according to a 12-h dark-light period. The rats were randomly divided into 4 groups, each group included 7 rats. Groups are control, AHA powder, AHA liquid, and Floseal.

**Figure 1.**
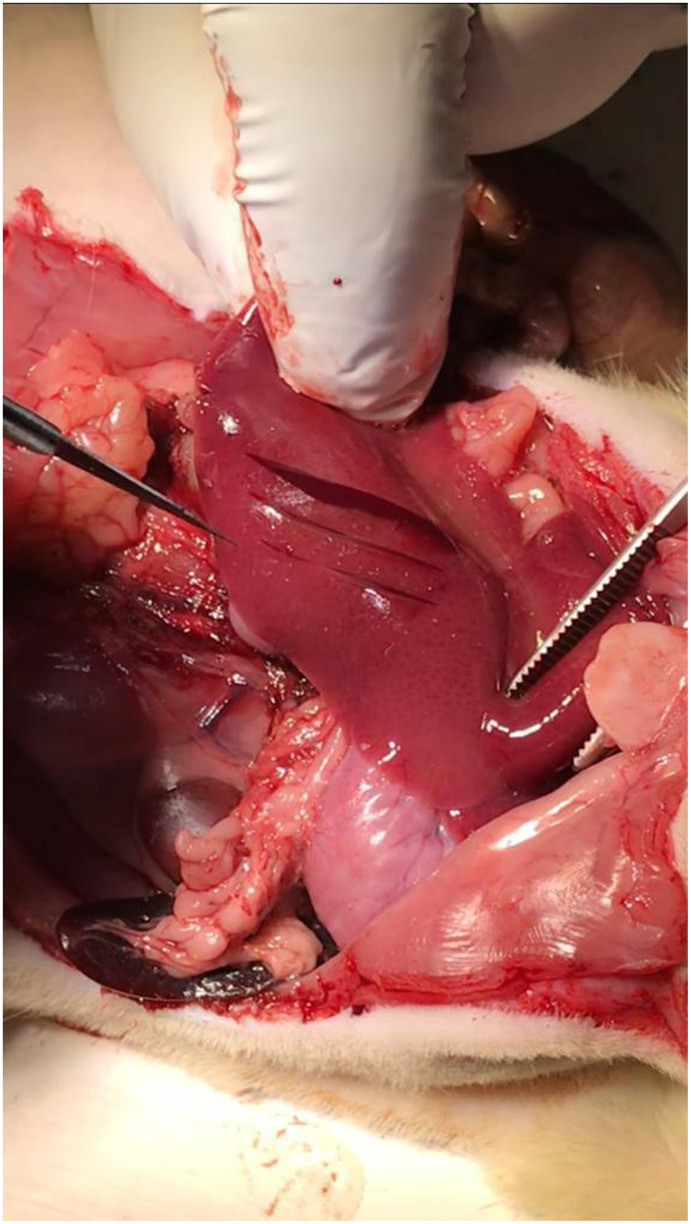
Creation of laceration in the liver.

Algan hemostatic agent was obtained from the Algan Group Health Services Import and Export Industry and Trade Limited Company (Istanbul, Turkey), and Floseal was obtained from the Baxter Healthcare Corporation (Deerfield, Illinois, U.S). To perform the experiment, the abdomen was opened 3 cm with the midline intersection. Following the opening of the animal’s peritoneal cavity, a total of 3 iatrogenic lacerations, 1 cm in length and 2 mm in depth, were implanted in the left lobe of the liver anteriorly (Figure 1). Procedures were performed under general anesthesia with ketamine hydrochloride (100 mg/kg, Pfizer Ltd. Şti.) and xylazine hydrochloride (10 mg/kg, Randlab Pty. Ltd, New York, ABD). At the end of the study, rats were euthanized as suggested in our previous study with 100 mg/kg intravenous sodium thiopental (Pental Sodium®, U.E. Ulagay, Istanbul, Turkey).

### Bleeding Test

The study was carried out as specified in the literature.^7^ In this study, a hemostatic agent was applied to the laceration area and no compression was applied on it. Bleeding time was measured. In another study, compression was employed for 3 minutes after a hemostatic agent was applied for liver laceration.^8^ In our study, mild compression was applied after the hemostatic agent was administered. Since it is known from previous trials and studies that AHA controls bleeding from liver laceration in about 10 seconds, 3 minutes was considered a long period. Therefore, in this study, hemostatic evaluation was performed as suggested in our previous study.^9^

**Figure 2.**
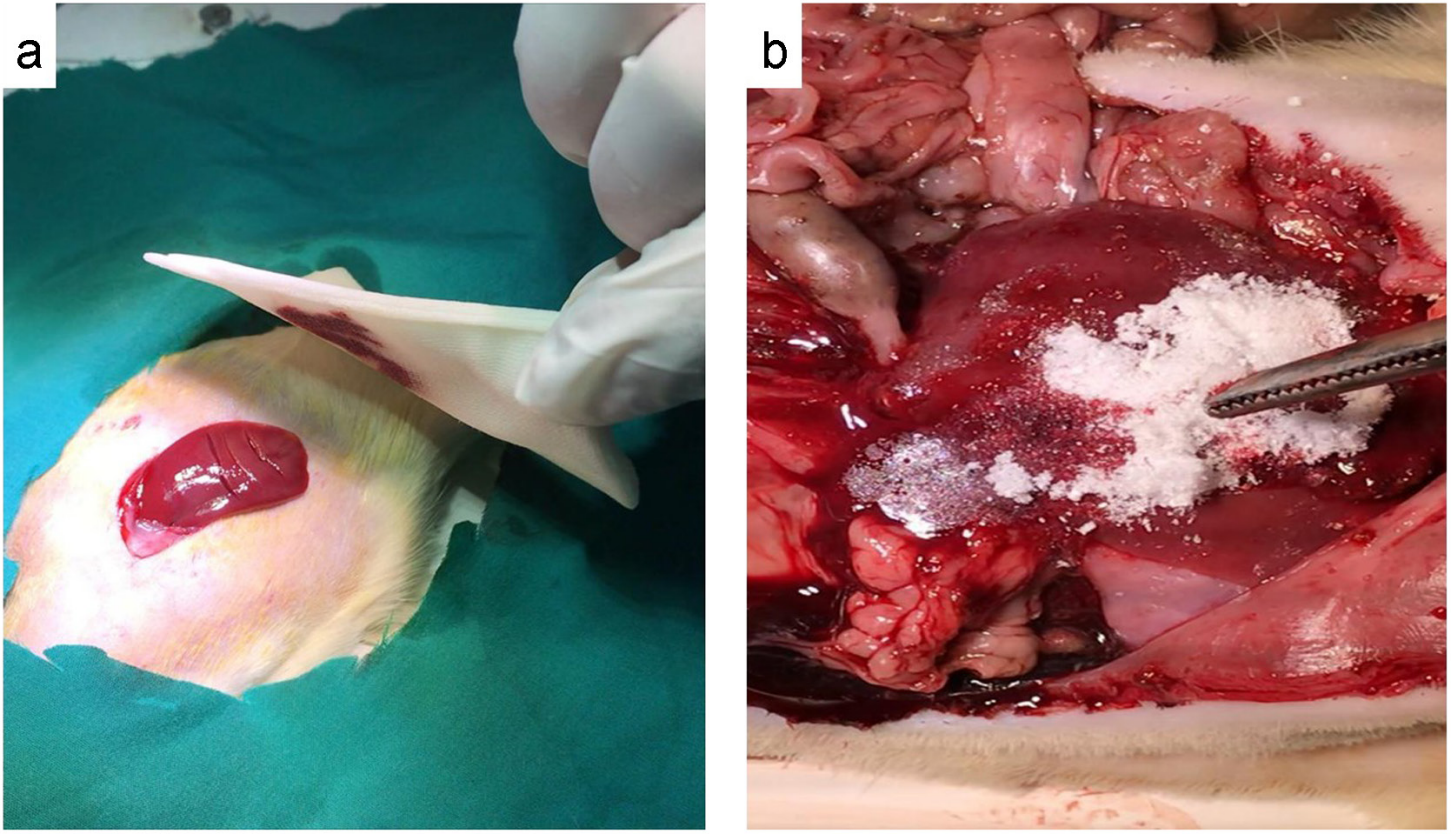
(A) AHA fluid in sponge. (B) AHA powder application. AHA, Algan hemostatic agent.

**Figure 3.**
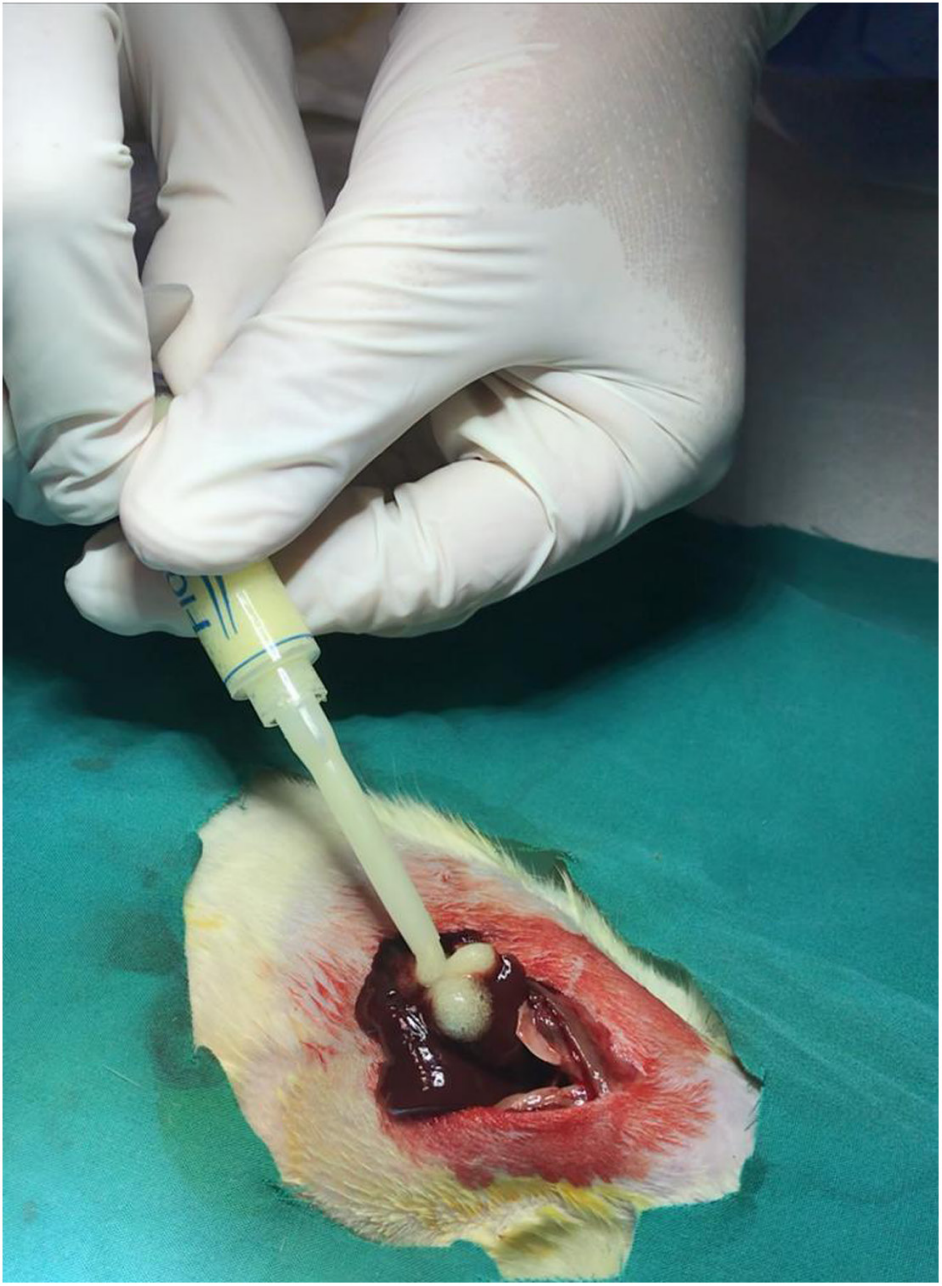
Floseal application.

After bleeding started, 2 cm^3^ of AHA fluid-impregnated sponge (Figure 2A), AHA powder (Figure 2B), Floseal (Figure 3), and saline solution-impregnated sponge were applied to the liver surface. Algan hemostatic agent powder was spread directly to the bleeding surface manually and not pressed on. The liquid form was utilized directly to the bleeding surface by means of a liquid-impregnated sponge and pressed on lightly. In case of the continuation of the bleeding, the procedure was repeated with the same amount of product. The first application lasted 15 seconds, the second application lasted 30 seconds, and the third and subsequent applications continued for 1 minute because it is known that AHA can control the bleeding in about 10 seconds.^22^ The application was measured by chronometry. After the procedure, hemostasis was observed in each group for 10 minutes.

## Statistical Analysis

Statistical Package for the Social Sciences software version 22.0 (IBM SPSS Corp.; Armonk, NY, USA) was used to analyze the data of this study. Bleeding time was calculated and mean values were compared among the 4 groups using Kolmogorov–Smirnov test. The results were assessed at a 95% CI and a significance level of *P* <.05.

## Results

Algan hemostatic agent powder and Floseal controlled the bleeding in all rats at first application. Bleeding could not be controlled in the control group. Algan hemostatic agent sponge controlled the bleeding in 6 rats at the first application and 1 rat at the second application. Results of control group were statistically different from the AHA powder, AHA liquid, and Floseal (*P* < .05). It was found statistically significant that AHA liquid, AHA powder, and Floseal controlled the bleeding in the first 15 seconds, and there was no statistical difference between these 3 products (*P* > .05). The success of the methods were compared with the Kolmogorov-Smirnov test. These results are disclosed in Table 1, and statistical findings are given in Table 2.

## Discussion

In this study, 2 different forms of AHA, powder and liquid (sponge), were compared with Floseal. All were found to be very effective. Although the powder form controlled bleeding more effectively, the fluid form of AHA was also effective and no statistically significant difference was observed between them in terms of bleeding control efficacy. However, when the control group was involved, a statistically significant difference was found.

The duration of bleeding in the control group was much longer than that of the experimental groups. The liquid form has promised to be an effective hemostat in internal bleeds that cannot be compressed.

In the literature, there are some studies comparing local hemostatic agents with similar studies.^7,8^ Although the incision techniques were similar, there were differences in intraoperative evaluations. In some studies, the hemostatic effects were evaluated by measuring the preoperative and postoperative hematocrit values and collecting bleedingG.^7,8,10,11^ In the last decade, various effective hemostatic agents have been designed in conjunction with conventional hemostatic techniques such as cauterization, direct pressure, and ligation.^5,6,12-18^ However, they have some limitations, such as inducing allergic reactions, unknown infections, and adhesion.^19, 20^

In some studies in the literature, Floseal was used as a comparison product in the evaluation of hemostatic efficacy.^12-18^ In many studies, Floseal® was shown to be more effective in reducing blood loss compared to other products.^12,13,17^ On the other hand, in a study of 80 patients, it was shown that Floseal did not provide an additional benefit to conventional hemostasis (electrocoagulation) in reducing blood loss in minimally invasive total knee arthroplasty.^21^ In the literature, there is a study comparing Floseal with others. In this study, control, Glubran 2, Celox, Floseal, and Ankaferd were compared. Partial nephrectomy was applied to rats. In this study, the best results were obtained with Glubran 2, while Floseal and Celox showed similar positive results.^5^ There are studies in the literature showing the effectiveness of Floseal in the liver hemorrhage model.^12,14,16^

Since the AHA powder form is easily absorbed, it is quickly cleaned from the area where it is was applied. In this way, it provides a clean working area in surgical operations. No side effects have been encountered in preclinical studies related to AHA.^22-26^

There are some commercial products that yield results similar to AHA. However, these commercial products have a critical disadvantage of nonconformity for deep and narrow wounds and they may cause tissue injury due to the large exothermic reaction.^27^ The majority of hemorrhage-related deaths in the military and civilian populations occur from non-compressible and heavy intra-cavitary hemorrhage that cannot be completely managed by tourniquets, external dressings, and bandages.^28,29^

The ideal hemostatic agent should be inexpensive, safe, effective, simple to apply, and should not be easily affected by environmental conditions. It must not allow the transmission of bacterial or viral infection, and it must be able to sustain hemostasis for at least several hours.

Algan hemostatic agent meets all of these requirements. In the application of the liquid form, no compression was applied to the area where it was applied after liver laceration, and hemostasis was achieved in 6 (86%) rats in the first 2 minutes of application.

Algan hemostatic agent is not expected to control bleeding effectively without compression after application in cases such as battlefield areas, traffic accidents, and severe physical injuries, where blood pressure is high and there is large-scale arterial bleeding. In these cases, AHA-impregnated sponges or AHA in powder form are applied to the bleeding area and compression is implemented. In addition, the fluid form of AHA can be practically utilized and is effective in bleeding control in cases of internal bleeding that cannot be compressed.

According to the results of this study, although the AHA is a highly effective hemostatic agent when used for this purpose in the liver laceration hemorrhage model in the literature, the actual difference can be demonstrated by more comparative studies. This situation will be clearer with future investigations.

As a result, AHA showed 100% success in controlling bleeding in the first 2 minutes like Floseal. The liquid form of AHA achieved 86% success in bleeding control in the first 2 minutes. These results show that AHA is a strong hemostatic candidate.

### Limitations of the Study

Although it is a standard procedure, the difference in the amount of bleeding among rats was one of the limitations of this study. In addition, there was a difference in form between the products used as liquid and powder.

Mount of intraoperative bleeding preoperative and postoperative hematocrit levels were not measured and hence their omission constitute another limitation of this study.

The low number of rats and histopathological examination can also be considered as another limitation of the study.

In conclusion, the efficacy of AHA in liver laceration bleeding model was compared with Floseal, which is widely used in the clinics and whose hemostatic efficiency is well known. The powder form of AHA was successful in controlling bleeding in all rats in the first application (15 seconds). Since similar results are observed in Floseal, studies with larger series or using different methods are required for superiority assessment. Although this study shows that AHA is a powerful hemostatic agent, it needs to be demonstrated with more independent studies.

## Figures and Tables

**Table 1.** Control and Homeostasis Times of Groups

	Bleeding Controlled at First Application (15 seconds)	Bleeding Controlled at First Application (30 seconds)	Bleeding Controlled at Thirth Application (60 seconds)	Unsuccessful
Control	0 (0%)	0 (0%)	0 (0%)	8 (100%)
AHA powder	7 (100%)	0 (0%)	0 (0%)	0 (0%)
AHA liquid	6 (86%)	1 (14%)	0 (0%)	0 (0%)
Floseal	7 (100%)	0 (0%)	0 (0%)	0 (0%)

AHA, Algan hemostatic agent.

**Table 2.** The Effect of AHA Fluid, AHA Powder, and Floseal on Bleeding in the First 15 Seconds

	P*
Control—AHA powder	.001
Control—AHA liquid	.012
Control—Floseal	.002
	
AHA powder—AHA liquid	.999
AHA powder—Floseal	1.000
AHA liquid—Floseal	.999

*Kolmogorov–Smirnov test.
